# Coping strategies among healthcare professionals facing the death of elderly patients in palliative care

**DOI:** 10.1192/j.eurpsy.2026.11645

**Published:** 2026-07-31

**Authors:** J. Ben Khelifa, C. Sridi, I. Kacem, N. Gannoun, N. Belhadj, F. Chelly, R. Nakhli, A. Fki, M. Maoua

**Affiliations:** 1 Clinical Psychology Unit of Sahloul University Hospital; 2 Occupational Medicine Department, Sahloul University Hospital; 3 University of Sousse, Faculty of Medicine of Sousse; 4 Research Laboratory LR19SP03; 5 Occupational Medicine Department, Farhat Hached University Hospital, Sousse, Tunisia

## Abstract

**Introduction:**

Nurses in palliative care are frequently exposed to emotionally demanding situations, which can lead to high levels of stress and an increased risk of burnout. Investigating the coping strategies they adopt is crucial for understanding how they manage these challenges and for developing interventions that promote their well-being.

**Objectives:**

This study aimed to describe the coping mechanisms used by healthcare professionals when confronted with the death of elderly patients in geriatric palliative care.

**Methods:**

A descriptive cross-sectional study was conducted with an analytical approach between November and December 2024. The sample consisted of 100 nurses working in the palliative care units of Sahloul University Hospital and Farhat Hached University Hospital. Data were collected using a self-administered questionnaire, the Burnout Scale, and the Coping Scale.

**Results:**

More than half of the participants (52%) reported sometimes feeling helpless when facing the death of an elderly patient. Avoidance was the most frequently reported defense mechanism (50%). Following the death of a patient, 52% of participants described experiencing anxiety and stress. Peer exchange and mutual support were identified as coping strategies by 54% of nurses. Additionally, 74% emphasized the need for specific training in managing suffering and negative emotions related to death in geriatric care. Regarding coping styles, the mean score for problem-focused coping (26.76 ± 5.38) was higher than that for emotion-focused coping (23.32 ± 4.30). A significant correlation was observed between the “problem-solving” dimension and length of service (p = 0.002).

**Conclusions:**

Coping strategies play a central role in stress management and burnout prevention among palliative care nurses. Our findings highlight the importance of providing targeted training in emotional regulation and stress management to better support healthcare professionals working in geriatric palliative settings.

**Disclosure of Interest:**

None Declared

